# Self-reported benzodiazepine use among adults with chronic spinal cord injury in the southeastern USA: associations with demographic, injury, and opioid use characteristics

**DOI:** 10.1038/s41393-024-01030-4

**Published:** 2024-09-13

**Authors:** Nicole D. DiPiro, Clara E. Dismuke-Greer, James S. Krause

**Affiliations:** 1 College of Health Professions, Medical University of South Carolina, Charleston, SC USA; 2 Health Economics Resource Center, Veterans Affairs Palo Alto, Menlo Park, CA USA

**Keywords:** Drug regulation, Risk factors

## Abstract

**Study design:**

Cross-sectional cohort study.

**Objectives:**

To examine: (1) the self-reported frequency of specific prescription benzodiazepine use, (2) concurrent benzodiazepine and opioid use, and (3) sociodemographic, SCI, and opioid use factors associated with frequent benzodiazepine use.

**Setting:**

Community.

**Methods:**

Participants included 918 community dwelling adults with chronic ( > 1 year) traumatic SCI originally identified from a specialty hospital or a state-based surveillance system. Self-reported frequency of specific prescription benzodiazepines and opioids used, concurrent use, and factors associated with use were assessed.

**Results:**

Twenty percent reported any benzodiazepine use in the past year and 13% reported at least weekly use. Concurrent daily or weekly use of benzodiazepines and opioids was reported by 6.5%, with those individuals taking an average of 1.1 (0.4) benzodiazepines and 1.4 (0.6) opioids. Compared to younger adults, those 50–65 years old had lower odds of at least weekly benzodiazepine use (OR = 0.50, 95% CI, 0.29–0.89, *p*-value = 0.02). Non-Hispanic Blacks reported lower use of benzodiazepines compared to non-Hispanic whites (OR = 0.32, 95% CI, 0.15–0.68, *p*-value = <0.01). Weekly opioid use was associated with higher odds of using benzodiazepines (OR = 3.10, 95%CI, 1.95–4.95, *p*-value = <0.01).

**Conclusions:**

Benzodiazepine use was commonly reported among those with SCI. Despite the potential risks, a high portion of those who reported benzodiazepine use also reported prescription opioid use. The findings highlight the need for monitoring of prescription medication use to avoid potentially risky concurrent use and adverse outcomes.

## Introduction

Individuals with spinal cord injury (SCI) experience an array of motor, sensory, and autonomic impairments, and are at increased risk of developing secondary and chronic health conditions, such as pain, spasticity, anxiety, and depression. The management and treatment of these complications and health conditions may involve long-term use of prescription medications, including pain relievers, tranquilizers, and sedatives. Furthermore, the complexity of SCI care often increases the risk of being prescribed or using two or more medications within the same time period, defined as concurrent, concomitant, or co-medication use [1]. To date, there has been limited study of specific prescription medications used by those with chronic SCI, and the prevalence of co-medication is not well understood. Although benzodiazepines and opioids are two commonly prescribed classes of medications in the United States (US) [2, 3] and among those with SCI [4–8], there is a paucity of data regarding the prevalence of and factors related to benzodiazepine use, and the rates of high-risk concurrent opioid use.

Benzodiazepines are a class of controlled drugs that exert sedative, hypnotic, or anxiolytic effects and are often used to treat anxiety and insomnia, as well as for muscle relaxation and spasticity; they may be described as tranquilizers or sedatives [9]. Benzodiazepines are among the most widely prescribed drugs in the US, and pose risks with long-term use, including adverse side effects, tolerance, dependence, misuse, overdose and death [9, 10]. Although benzodiazepines have been reported to be commonly used after SCI [4, 5, 11], there has been limited research. One study of adults with non-traumatic SCI found that 29% of participants had a benzodiazepine dispensed in the year after inpatient rehabilitation [11]. Another recent study found that among those with SCI, short-acting benzodiazepines were prescribed for spasticity outside the recommended treatment paradigm at a high rate [12]. To date, we have little understanding of specific benzodiazepines used, the prevalence and frequency of use, and the factors that may influence benzodiazepine use among those with chronic SCI.

A major area of concern is the concurrent use of benzodiazepines and opioids. Because both drugs depress the central nervous system, when used concurrently, there is an increased risk of respiratory depression, emergency department utilization, drug-related emergency admissions, overdose, and death [3, 13–15]. These adverse events may occur in both those who do and do not abuse drugs. Although published guidelines advise clinicians to consider the risks and benefits, and avoid co-prescribing benzodiazepines and opioids when possible [16], the prevalence of concurrent use of these drugs among Americans is notable and a driver of the current opioid crisis. Studies examining data from the early 2000s–2010s found benzodiazepine and opioid co-medication rose significantly within the general US population, contributing to the rise in overdose and deaths [3, 13, 14]. More recent reports suggest there was a decline in concurrent use between 2017 and 2018, with roughly 14% of opioid users also on a benzodiazepine [17].

Within the SCI population, high-risk concurrent use of benzodiazepines and opioids has been noted [6, 18]. Using a state prescription drug monitoring program to examine opioid use in years 2–3 after SCI, it was found that 38% of individuals who filled a prescription for an opioid had a concurrent fill for a benzodiazepine, and of those, one quarter had a drug overlap over 360 days [6]. In a later assessment of high-risk use, it was noted that of the concurrent benzodiazepine, sedative and hypnotic fills, the majority (76%) were for benzodiazepines [18].

Considering the limited study of benzodiazepine use after SCI, the high rate of concurrent use previously reported, and the risks associated with using both benzodiazepines and opioids, there is a need for continued study. Our purpose was threefold: (1) examine the self-reported past year frequency of use of specific prescription benzodiazepines, (2) identify the frequency of concurrent benzodiazepine and opioid use, and (3) identify the factors associated with frequent benzodiazepine usage, including demographic, SCI, socioeconomic characteristics, and opioid usage.

## Methods

### Participants and data sources

Institutional review board approval was obtained prior to initiating the study procedures. Eligibility criteria included: adults ( > 18 years old) with chronic ( > 1 year post injury), traumatic SCI with non-complete recovery. Individuals were recruited from a pool of participants from longitudinal studies of health outcomes after SCI. Originally, the participant cohorts were identified from a regional specialty hospital in the southeastern United States; the first cohort enrolled in 1997–1998 and the second enrolled in 2007–2010. An additional cohort was identified from a state-based surveillance system in the southeastern United States and added in 2012–2016. All participants were previously enrolled in follow-up studies of persons with SCI. For the current study, 1407 participants were invited to participate. There were a total of 918 respondents (65.2% response rate), 25.5% of whom were from the state-based surveillance system.

### Data collection procedures

In 2021–2022, potential participants were sent cover letters explaining the study and informing them of forthcoming study materials. The cover letter contained a link to the online REDCap self-report assessment (SRA), which allowed participants to complete the materials virtually. A paper form of the SRA was mailed 4–6 weeks later for all individuals who did not complete the materials in REDCap. A second mailing was used to promote response, followed by a phone call from the study coordinator. For individuals who agreed to participate but had discarded or misplaced the SRA, another set of materials were mailed. Return of completed materials implied consent, and a waiver of signed consent was obtained from the IRB. Participants were offered $50 in remuneration.

### Measures

The SRA included items on demographic characteristics (age, race-ethnicity, sex), SCI characteristics (injury severity, years since onset), socioeconomic indicators (education and income), and prescription medication items from the National Survey on Drug Use and Health (NSDUH) [19]. We identified self-report of benzodiazepines use based on the tranquilizer and sedative screeners, as well as self-reported prescription pain relievers (opioid) use.

Age was broken down into three categories: <50, 50–64, ≥65. Race-ethnicity was grouped as follows: non-Hispanic White, non-Hispanic Black, and Hispanic and non-Hispanic other. Neurologic level was broken down into four categories, the first of which was all ambulatory, and the other three categories broken down according to neurologic level as follows: high cervical (C1–C4), low cervical (C5–C8), and noncervical. Years since injury was broken down into the following categories: 1–9 years, 10–19 years, and ≥20 years. Years of education was broken down into three levels: high school diploma/GED or less, trade school/associate’s degree, and bachelor’s degree or higher. Family income was broken down into three levels: <$25,000, $25,000–74,999, and > $75,000.

To assess prescription benzodiazepine use, participants were asked, “In the past 12 months, which, if any, of these sedatives or tranquilizers have you used?” and asked to indicate the frequency of use as Never, Once or twice, Monthly, Weekly, or Daily/almost daily. The SRA informed them that “tranquilizers are usually prescribed to relax people, to calm people down, to relieve anxiety, or to relax muscle spasms; and that some people call them nerve pills.” Prescription sedatives were defined as “drugs that are also called sleeping pills or downers. People take these drugs to help them relax or help them sleep.” The specific benzodiazepines included: (a) Alprazolam (Xanax®, Xanax® XR); (b) Lorazepam (Ativan®); (c) Clonazepam (Klonopin®); (d) Diazepam (Valium®); (e) Flurazepam (Dalmane®); (f) Temazepam (Restoril®); and (g) Triazolam (Halcion®).

For the opioid items, participants were asked, “In the past 12 months, which, if any, of these prescription pain relievers have you used?” and reported the frequency of use (Never, Once or twice, Monthly, Weekly, or Daily/almost daily). Participants were asked about their use of prescription pain relievers, because they were more likely to understand the term “pain relievers” rather than “opioids,” since “pain relievers” indicates the purpose for which the drugs are likely to be taken. Eleven common opioids comprise the item set. The methods of assessment have been documented in a previous manuscript [20].

Based on participant responses and regularity of use, we combined “once or twice” with “monthly” use, and “daily/almost daily” use with “weekly” use; we refer to once/monthly or weekly/daily use of the drugs in the cross-tabulation and the logistic regression model. Our operational definition of *concurrent use* included those individuals who indicated “weekly” or “daily/almost daily” using both any opioid and any benzodiazepine. Although individuals who indicated using these medications “monthly,” or “once or twice,” may also experience risky concurrent use, due the greater uncertainty of overlap in the frequency of use, they were not included in our definition.

### Data analysis

Missing data were examined and handled based on two criteria: First, if an individual skipped all of the specific drugs in the benzodiazepine or opioid modules, they were classified as *missing* (*n* = 13 (1.4%) were missing all benzodiazepine items, and *n* = 17 (1.9%) were missing all opioid items. Among those 30 individuals, 4 skipped both sections; in total, 26 individuals were missing benzodiazepines or opioids and excluded from the prescription specific descriptive analyses, therefore we report *n* = 905 for the presentation of benzodiazepine use groups, and *n* = 892 for the cross-tabulation of use). Second, if an individual answered one or more specific drugs in either module, while leaving other drugs blank, those missing drugs were assumed to be “no/never use,” and classified as such.

STATA Version 16.1 was used for all statistical analyses. First, we examined the frequencies of use of each prescription medication, then categorized use of benzodiazepines and opioids into any use (yes/no), once/monthly use (yes/no), and weekly/daily use (yes/no). Because participants may report taking more than one prescription benzodiazepine or opioid, they may fall into both categories. We also assessed concurrent use by defining those who responded “yes” to weekly/daily opioid and benzodiazepine usage. Descriptive statistics (*n*, %) and a contingency table of frequency of use are presented.

The relationships between each variable and the dependent variable (weekly/daily benzodiazepine use) were analyzed. We ran a simple logistic regression and a full multiple logistic regression model of binary indicators of weekly/daily benzodiazepine use (yes/no) using logit model estimation methods. The full model included age, years since injury, SCI injury severity, race/ethnicity, Military Veteran status, education, household income, and weekly/daily opioid use as covariates. Odds ratios (OR) and 95% confidence intervals are reported.

## Results

### Participant characteristics

Full sample participant demographics and injury characteristics (*n* = 918), and characteristics by benzodiazepine use groups (*n* = 905) are presented in Table 1; the 13 individuals who skipped all benzodiazepine questions were not included in the group specific descriptive statistics. The majority of the participants were male (69%), non-Hispanic white (70%), and younger than 65 (70%). The participants were on average 24.4 (10.7) years post-injury, with a range of 2–75 years post-injury. Forty-four percent were ambulatory. The remainder of participants were non-ambulatory, with 7% reporting high cervical (C1–C4), 20% low cervical (C5–C8), and 29.4% non-cervical injuries.

**Table 1:** Demographic and participant characteristics within the full sample (*n* = 918) and benzodiazepine use groups (*n* = 905) (*n*, %).

		Past 12 Month Benzodiazepine Use Groups^a^		
Participant Characteristics	Full Sample *n* = 918	None *n* = 725	Once/monthly use *n* = 62	Weekly/daily use *n* = 118
Age				
<50	254 (27.7%)	192 (26.5%)	25 (40.3%)	37 (31.4%)
50–64	387 (42.2%)	306 (42.2%)	27 (43.5%)	47 (39.8%)
≥65	277 (30.2%)	227 (31.3%)	10 (16.1%)	34 (28.8%)
Years post-injury				
2–14 years	115 (12.5%)	93 (12.8%)	9 (14.5%)	12 (10.2%)
15–29 years	560 (61.0%)	451 (62.2%)	34 (54.8%)	69 (58.5%)
≥30 years	243 (26.5%)	181 (25.0%)	19 (30.6%)	37 (31.4%)
Race-ethnicity				
Non-Hispanic White	639 (69.7%)	484 (66.8%)	50 (80.6%)	100 (85.5%)
Non-Hispanic Black	229 (25%)	203 (28.0%)	7 (11.3%)	12 (10.3%)
Hispanic/Other	49 (5.3%)	38 (5.2%)	5 (8.1%)	5 (4.3%)
Injury level				
C1–4	62 (7%)	46 (6.5%)	4 (6.5%)	11 (9.7%)
C5-C8, non-ambulatory	179 (20.1%)	138 (19.6%)	12 (19.4%)	27 (23.9%)
Non-cervical, non-ambulatory	262 (29.4%)	210 (29.9%)	17 (27.4%)	32 (28.3%)
Ambulatory	388 (43.5%)	309 (44.0%)	29 (46.8%)	43 (38.1%)
Veteran (yes)	112 (12.3%)	96 (13.3%)	5 (8.1%)	8 (6.8%)
Education				
HS diploma/GED or less	360 (40.1%)	286 (40.2%)	22 (35.5%)	43 (37.4%)
Associate’s/two-year degree	243 (27.1%)	188 (26.4%)	20 (32.3%)	34 (29.6%)
Bachelor’s or higher	295 (32.9%)	237 (33.3%)	20 (32.3%)	38 (33.0%)
Household income				
<$25,000	394 (45.2%)	303 (43.8%)	34 (55.7%)	52 (46.4%)
$25,000–$74,999	285 (32.7%)	236 (34.2%)	14 (23.0%)	34 (30.4%)
>$75,000	192 (22.0%)	152 (22.0%)	13 (21.3%)	26 (23.2%)

^a^*n* = 905; 13 cases skipped all questions related to self-reported benzodiazepine use, therefore were classified as missing, and omitted from the group classifications.

### Frequency of use of specific benzodiazepines, opioids, and concurrent use

Overall, of the 918 participants, 19.6% (*n* = 180) reported any past-year benzodiazepine use (indicating use of at least one prescription benzodiazepine), with 7.1% (*n* = 65) reporting infrequent, once or monthly use, and 12.9% (*n* = 118) reporting frequent use, at least weekly; three individuals were categorized into both once/monthly and weekly/daily groups based on the report of using more than one benzodiazepine, with differing frequencies of use. Nearly 50% of the sample reported any past year use of opioids (*n* = 432), with 22.7% (*n* = 227) indicating once/monthly use and 28.8% (*n* = 264) indicating weekly/daily use; because individuals may report using more than one opioid, with differing frequencies of use, 59 individuals were categorized into both once/monthly and weekly/daily groups.

The specific prescription benzodiazepine and opioid medications and reported frequency of use are presented in Table 2. Within the full cohort, Diazepam was the benzodiazepine most frequently reported at the daily/almost daily level (4%), followed by Alprazolam (3%). Oxycodone was the most frequently used daily/almost daily opioid (10%).

**Table 2:** Frequency of use of specific benzodiazepines and opioids, *n* = 918.

	*N* (%)				
	Never	Once or twice	Monthly	Weekly	Daily/almost daily
**Benzodiazepines**					
*Alprazolam*	837 (92.5)	29 (3.2)	4 (0.4)	8 (0.9)	27 (3.0)
*Lorazepam*	883 (97.6)	7 (0.8)	3 (0.3)	3 (0.3)	9 (1.0)
*Clonazepam*	871 (96.2)	9 (1.0)	3 (0.3)	7 (0.8)	15 (1.7)
*Diazepam*	839 (92.7)	14 (1.5)	7 (0.8)	9 (1.0)	36 (4.0)
*Flurazepam*	901 (99.6)	3 (0.3)	0 (0)	0 (0)	1 (0.1)
*Temazepam*	897 (99.1)	5 (0.6)	0 (0)	1 (0.1)	2 (0.2)
*Triazolam*	896 (99.0)	1 (0.1)	2 (0)	2 (0.2)	4 (0.4)
**Opioids**					
*Hydrocodone*	713 (79.1)	89 (9.9)	14 (1.6)	24 (2.6)	61 (6.8)
*Oxycodone*	734 (81.5)	54 (6.0)	7 (0.8)	13 (1.4)	93 (10.3)
*Tramadol*	769 (85.3)	61 (6.8)	11 (1.2)	13 (1.4)	47 (5.2)
*Codeine*	826 (91.7)	43 (4.8)	13 (1.4)	11 (1.2)	8 (0.9)
*Morphine*	852 (94.6)	22 (2.4)	2 (0.2)	2 (0.2)	23 (2.6)
*Fentanyl*	880 (97.7)	11 (1.2)	1 (0.1)	1 (0.1)	8 (0.9)
*Buprenorphine*	887 (98.4)	2 (0.2)	3 (0.3)	3 (0.3)	6 (0.7)
*Oxymorphone*	896 (99.4)	1 (0.1)	0 (0)	1 (0.1)	3 (0.3)
*Meperidine*	896 (99.4)	4 (0.4)	1 (0.1)	0 (0)	0 (0)
*Hydromorphone*	882 (97.9)	7 (0.8)	2 (0.2)	1 (0.1)	9 (1.0)
*Methadone*	888 (98.6)	3 (0.3)	2 (0.2)	0 (0)	8 (0.9)

*N* = 13 (1.4%) were missing ALL Benzodiazepine data. The frequencies shown are valid percent for those with data, summing to 100%.

*N* = 17 cases (1.9%) were missing ALL opioid data. The frequencies shown are valid percent for those with data, summing to 100%.

A crosstabulation of the frequency of benzodiazepine and opioid use (*n* = 892), broken into three categories (none, once or monthly, and weekly or daily) is presented in Table 3. Individuals who skipped all benzodiazepine (*n* = 13) and all opioid questions (*n* = 17) were omitted from the group classifications; four of those individuals were missing both opioids and benzodiazepines, therefore 26 individuals were omitted from the contingency table. The frequency of use of benzodiazepines was associated with opioid use frequency (X^2^ (4) = 52.8, <0.001, φ = 0.243).

**Table 3:** Contingency table of self-reported reported benzodiazepine and opioid use, *n* = 892^a^.

	No Opioid Use	Once/Monthly Opioid Use	Weekly/Daily Opioid Use	Total
**No Benzodiazepine Use (*n*)** *% within Benzodiazepine groups*	409 57.3%	120 16.8%	185 25.9%	714
**Once/Monthly Benzodiazepine Use (*n*)** *% within Benzodiazepine groups*	19 30.6%	24 38.7%	19 30.6%	62
**Weekly/Daily Benzodiazepine Use (*n*)** *% within Benzodiazepine groups*	37 31.9%	21 18.1%	58 50%	116
**Total** *% within Benzodiazepine groups*	465 52.1%	165 18.5%	262 29.4%	892

Pearson Chi-Sq (X^2^ (4) = 52.8, <0.001).

φ = 0.243 ( < 0.001).

^a^26 individuals who were missing all opioid data or all benzodiazepine data were excluded (17 cases skipped all opioid questions and 13 skipped all benzodiazepine; among those 30 individuals 4 skipped both sections).

Row percentages are presented.

Examining dual and concurrent use, we found 13.5% (*n* = 122) reported any past year use of both benzodiazepines and opioids, and 6.4% (*n* = 58) reported frequent concurrent use (taking at least one benzodiazepine and one opioid at least weekly or daily in the past year). Among the 58 participants who reported weekly or daily concurrent use of opioids and benzodiazepines, the mean (SD) number of weekly or daily benzodiazepines used was 1.09 (0.38) with a range of 1–3 different benzodiazepines and the mean (SD) number of weekly or daily opioids used was 1.41 (0.62) with a range of 1–3 different opioids. Combined, the mean (SD) number of weekly prescription benzodiazepines or opioids was 2.50 (0.86), with a range of 2–6 medications.

### Regression analyses of weekly or daily benzodiazepine use

**Fig. 1:**
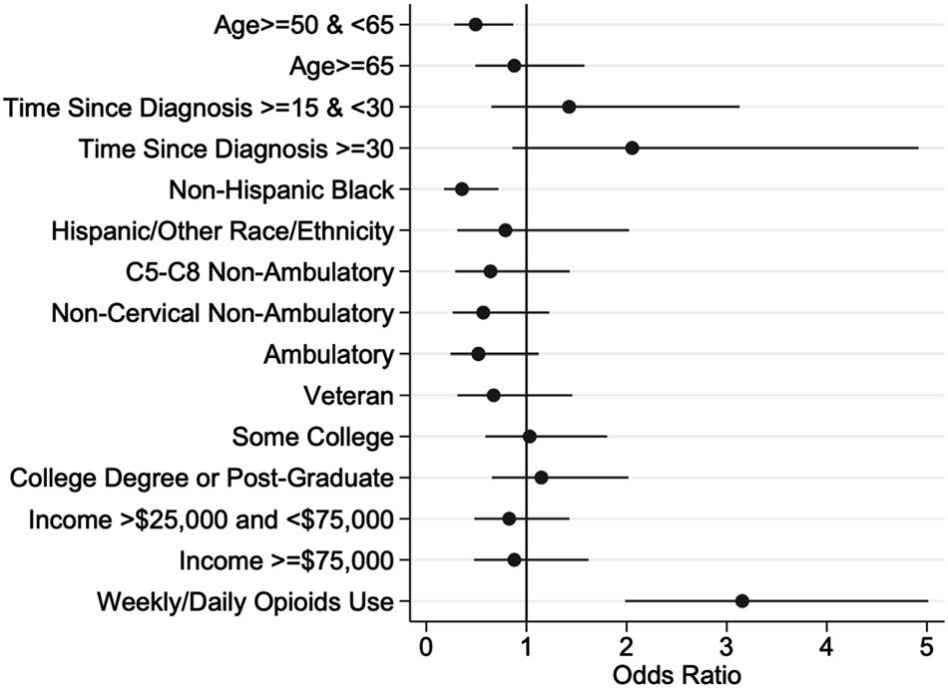
Forest plot of the factors associated with weekly or daily use of at least one prescription benzodiazepine within the past 12 months. Forest plot illustrating the odds ratios and 95% confidence intervals from a logit model predicting the odds of weekly or daily benzodiazepine use in the last 12 months. The odds ratios on the *x*-axis are greater than one if the value of a factor has a higher odds of benzodiazepine use in the last 12 months than the reference group for a particular factor, while those less than one have a lower odds of benzodiazepine use than the reference group for a particular factor. The closed dots represent the odds ratios while the lines on either side of the closed dots represent the 95% confidence intervals. The red line represents an odds ratio of 1.

In the baseline simple logistic analyses (Table 4), individuals who are non-Hispanic Black were less likely to report weekly or daily benzodiazepine use than non-Hispanic white adults (OR = 0.31, 95% CI, 0.17–0.59), and reporting weekly or daily opioid use was associated with a higher odds (OR = 2.8, 95% CI, 1.88–4.17) of weekly or daily benzodiazepine use. In the full multivariable model, two demographic factors were significantly related to weekly benzodiazepine use (Table 4, Fig. 1). Those aged 50–65 reported significantly lower use compared with those under 50 (OR = 0.50, 95% CI, 0.29–0.89, *p*-value = 0.02) and non-Hispanic Black reported lower use compared with non-Hispanic white participants (OR = 0.32, 95% CI = , 0.15–0.68, *p*-value = <0.01). Use of weekly or daily opioids was associated with significantly higher odds of using benzodiazepines weekly or daily in the past year (OR = 3.10, 95% CI, 1.95–4.95, *p*-value < 0.01).

**Table 4:** Factors associated with weekly or daily use of at least one prescription benzodiazepine within the past 12 months.

	Simple Logistic Regression Models				Multiple Logistic Regression Model^a^			
	Odds Ratio		95% CI^b^	*p*	Odds ratio	95% CI^b^		*p*
Age (ref: <50)								
50–64	0.81	0.51	1.29	0.38	0.50	0.29	0.89	**0.02**
≥65	0.83	0.50	1.38	0.47	0.89	0.49	1.60	0.69
Years post-injury (ref: 1–14 years)								
15–29 years	1.45	0.72	2.92	0.29	1.39	0.63	3.07	0.40
≥30 years	1.91	0.91	3.99	0.09	1.94	0.81	4.66	0.13
Race-ethnicity (ref: non-Hispanic White)								
Non-Hispanic Black	0.31	0.17	0.59	**<0.01**	0.32	0.15	0.68	**<0.01**
Hispanic/Other	0.66	0.25	1.71	0.39	0.79	0.31	2.03	0.63
Injury level (ref: C1-4)								
C5-C8, non-ambulatory	0.81	0.38	1.76	0.60	0.63	0.28	1.41	0.26
Non-cervical, non-ambulatory	0.63	0.30	1.34	0.23	0.56	0.25	1.21	0.14
Ambulatory	0.56	0.27	1.17	0.12	0.51	0.23	1.09	0.08
Veteran (yes)	0.51	0.24	1.07	0.08	0.61	0.27	1.37	0.23
Education (ref: HS diploma/GED or less)								
Associate’s/two-year degree	1.13	0.69	1.85	0.62	1.06	0.61	1.87	0.83
Bachelor’s or higher	1.07	0.67	1.71	0.78	1.19	0.67	2.09	0.56
Household income (ref: <$25,000)								
$25,000–$75,000	0.86	0.54	1.38	0.53	0.83	0.48	1.43	0.50
>$75,000	1.02	0.61	1.69	0.95	0.87	0.47	1.61	0.66
Weekly or Daily Opioids (yes)	2.80	1.88	4.17	**<0.01**	3.11	1.95	4.95	**<0.01**

^a^Multiple logistic regression (logit), Pseudo R^2^ = 0.08.

^b^CI confidence intervals.

## Discussion

This study addresses a gap in the literature related to prescription benzodiazepine use, and highlights the frequency of concurrent benzodiazepine and opioid use among individuals with chronic SCI. The roughly 20% of participants who reported any past year benzodiazepine use, while not directly comparable, is higher than annual rates reported in the general US population based similarly on data from the NSDUH (12.6%) [10]. In addition to addressing any past year use, as is done in the national dataset, we further examined usage patterns, finding that 13% of our cohort reported at least weekly benzodiazepine use, indicating that these drugs are frequently used, and for extended periods of time (over the past year). Although the duration of “long-term use” varies in the literature (e.g. ≥60 days, ≥90 days) [21], long-term use of benzodiazepines, is cautioned against, especially in older adults older than 65 years old [22, 23], as there are increased risks for tolerance, dependence, misuse, and overdose. In our sample, the mean age was 54.5, and 30% of participants were over 65, raising concerns over the potential risks.

In order to focus on those who may be at greatest risk for adverse outcomes, we identified those who reported both use of a benzodiazepine and an opioid, defining concurrent use as at least weekly use of a benzodiazepine and opioid within the past year, as opposed to any past year dual use. The rate of weekly or daily concurrent use within the whole cohort (6.5%) is concerning considering the potential for adverse outcomes, and the finding that the majority of those who reported past year benzodiazepine use also reported past year opioid use (68.5%) is alarming. 9Furthermore, using a less conservative definition of co-medication use (any combined use in the past year) the estimate of annual co-use of benzodiazepines and opioids increases to 13.5%. Within our cohort, the average number of benzodiazepine or opioid medications taken at least weekly was 2.6. This does not consider other common medications these individuals may frequently take for SCI related complications.

Individuals with SCI are often prescribed and take a large number of medications to treat and manage the array of chronic and secondary health conditions and complications they experience, often being prescribed medications from multiple high-risk classes, as well as multiple medications within each class [4, 24, 25]. Jensen et al. found that after a SCI, medication use increased over three times; among those with traumatic injury, there was a 4.7 times increase in medication consumption. A few studies have reported on the numbers of drugs taken and rates of *polypharmacy* (often defined as taking 5 or more concomitant medications), which are estimated to range from 31–87% [4, 11, 25, 26]. Guilcher et al. found that within a cohort of adults with traumatic SCI who were older than 66 year old, 56% were taking more than 10 drug classes, and an average of 11 drug classes taken post injury [4]. Polypharmacy after SCI increases the risk of experiencing negative impacts on health and quality of life, potentially harmful drug interactions, unintentional injuries including falls and overdose, and death, regardless of misuse of these medications [5, 24, 27, 28]. Better understanding the demographic and injury characteristics that are associated with medication use, and the associations between medication classes may help to not only direct clinicians in their care plans but also attenuate risk, and will provide new knowledge for guiding future research.

### Methodological considerations

There are several methodologic considerations. First, we examined the factors associated with at least weekly benzodiazepine use, focusing on frequent use, as opposed to a more broad outcome of any past year use as is used in national datasets. Second, all data were self-report. While we did not have access to prescription data (e.g. dose, duration) to more fully understand benzodiazepine usage, simply identifying the frequency of usage is beneficial to our understanding by reflecting actual use, rather than prescriptions claims where the individual may or may not use them as directed. Although we found minimal risk of underreporting in a preliminary investigation into self-reported medication use compared to state prescription drug monitoring data [29], there may be errors in reporting due to intentional or unintentional inaccurate reporting due to recall or not understanding prescription names. Because of the anonymous reporting, there is no reason to expect intentional misreporting in the study. Third, the participants were drawn from an ongoing longitudinal study, and there was some attrition or nonresponse (62.5% response rate). A previous study of these participants examined responders and non-responders finding that males and those with less time since injury were more likely to be non-respondents [30]. The non-response would be more likely to affect estimates of usage, compared with covariates of benzodiazepines including both demographic and opioid-related variables. Fourth, in terms of participant recruitment, we used a combination of participants identified from statewide population-based surveillance, which captures all civilian cases within a given region, and those identified through a model system of care in the Southeastern US. Our reported rates of benzodiazepine and opioid use may not be generalizable to those with SCI nationally, as prevalence rates are known to vary by state and region. Population-based surveillance is the gold standard for epidemiology in the determination of prevalence rates. Lastly, we used a conservative estimate of concurrent use by requiring at least weekly use; higher rates would be expected with less restrictive definitions.

### Future research

The current study is only one of the first steps towards an adequate understanding of benzodiazepine use and high-risk concurrent use of opioids among those with chronic SCI. Future studies of larger samples may allow for modeling of frequent concurrent use, or the number of concurrent benzodiazepine and opioid prescriptions individual report taking. Future study of prescription claims may help us better understand prescribing rates. Additional research should focus on the health conditions and factors related to their management and treatment that may lead to co-medication. There is also a need to better understand the associated risks of concurrent benzodiazepine and opioid use in this population, for example, the rates of falls, respiratory related outcomes, hospital utilization, overdose and death associated with co-medication. Studies are needed that more closely examine the consequences of each class of medication used individually or together, with an emphasis on the adverse outcomes.

## Conclusions

Benzodiazepines are a commonly used class of prescription medications among those with chronic SCI, and are most often taken on a weekly basis. Concurrent benzodiazepine and opioid use was detected in a small but notable number of participants. Despite guidelines against concurrent use and significant associated risks, we found weekly or daily opioid use in the past year was associated with a greater likelihood of weekly benzodiazepine use. The findings highlight the need for monitoring of prescription medication use to avoid risky concurrent medication use and potential adverse outcomes.

## Data Availability

The data sets generated and/or analyzed during the current study are not publicly available due to the privacy concerns of study participants but are available from the corresponding author on reasonable request.
